# Transcriptomic analysis of oxidative stress mechanisms induced by acute nanoplastic exposure in *Sepia esculenta* larvae

**DOI:** 10.3389/fphys.2023.1250513

**Published:** 2023-08-08

**Authors:** Xiumei Liu, Jianmin Yang, Zan Li

**Affiliations:** ^1^ College of Life Sciences, Yantai University, Yantai, China; ^2^ School of Agriculture, Ludong University, Yantai, China

**Keywords:** nanoplastics, oxidative stress, ribosome, proteasome, MAPK signaling pathway

## Abstract

Nanoplastics (NPs), as a new type of pollutant with a size small than 1 μm, are ubiquitous and harmful to organisms. There has been an increasing amount of research concerning the effects of NPs on organisms over recent years, especially on aquatic animals. However, there is a limited study on the impact of NPs on mollusk cephalopods. In this research, *Sepia esculenta*, belonging to Cephalopoda, Coleoidea, Sepioidea, was selected to explore the effects caused by NPs exposure. The *S. esculenta* larvae were exposed to polystyrene NPs (PS-NPs) with diameter 50 nm (100 mg/L) for 4 h. The detection of oxidative stress biomarkers displayed an obvious increase in SOD (superoxide dismutase) activity and MDA (malondialdehyde) level. Then, RNA-Seq was performed to explore the oxidative stress response at mRNA level. The transcriptome analysis demonstrated that the expression of 2,570 genes was affected by PS-NPs. Besides, the signaling pathways of ribosome, ribosome biogenesis in eukaryotes, proteasome, and MAPK were enriched. This study not only provides novel references for understanding the mechanisms of oxidative stress response induced by NPs, but also reminds us to follow with interest the influence of acute exposure to NPs.

## 1 Introduction

Plastics have been used heavily due to their incredible versatility, which have had a huge impact on society and environment (Porta, 2021). The growth rate of this material production is astonishing, with a production of approximately 390 million metric tons in 2021 (Statista Research Department, 2023). At present, the plastic industry has become one of the world’s largest manufacturing industries. It is estimated that plastic production will increase by more than twice the current production by 2100 (Stegmann et al., 2022). However, most of the plastic currently produced is disposable, resulting in a large amount of waste plastics. Due to the high price of recycled plastics, the vast majority of waste plastics are traditional plastics that are difficult to biodegrade. In addition, improper handling methods have led to the accumulation of most waste plastics in the environment (Geyer et al., 2017). This has made plastic pollution a significant concern for people. The common waste plastics are thermoplastics used for packaging, for instance, polystyrene (PS), polyvinyl chloride (PVC), and polyethylene (PE) (Narancic and O'Connor, 2019). Plastic pieces in the environment constantly break down through biological, chemical, and physical processes, ultimately decomposing into nanoscopic fragments, known as nanoplastics (NPs, <1 μm) (Gigault et al., 2018; Ferreira et al., 2019). According to ATSDR reports, PS and PVC are common NPs (Kumar et al., 2022).

NPs are widely distributed in various ecosystems, causing certain damage to organisms and even posing a threat to human health. Ingestion of aquatic organisms contaminated with NPs is one of the main routes of NPs exposure to humans (Leslie et al., 2022). Molluscs are one of the main aquatic foods that provide abundant nutrients for humans. However, NPs can lead to toxicity on mollusks just like other animals (Ferreira et al., 2019). First of all, NPs can reduce the successfully fertilization rate of gametes and increase the malformation rate of embryo-larval development (Tallec et al., 2018; Rist et al., 2019). Through multiple methods, such as determination of i-Ca and transcriptome analysis, the researchers found that NPs led to the growth inhibition of *Tetrahymena thermophile* by affecting Ca signaling, and phosphatidylinositol signaling (Wu et al., 2021). Transcriptome analysis results displayed that NPs hindered the reproduction and population growth of *Brachionus plicatilis* by causing metabolic abnormalities and oxidative stress (Shin and Jeong, 2022). In addition, it was found that NPs not only triggered the apoptosis in different tissues of *Corbicula fluminea*, but also induced intestinal epithelial inflammatory response (Li et al., 2021). Through multi-omics and histopathological analysis, it was found that NPs induced intestinal epithelial damage, intestinal microbial community changes, and abnormal metabolism of carbohydrates and arachidonic acid in *Eisenia fetida* (Tang et al., 2023). In the intestine of *Apostichopus japonicas* and *Procambarus clarkii*, the changes in microbial community caused by NPs exposure might be related to oxidative stress (Han et al., 2022; Zhao et al., 2023). Oxidative stress is the common response caused by NPs in mollusks, such as *Mytilus* spp. (Cole et al., 2020), *C. fluminea* (Li et al., 2020), *Mytilus galloprovincialis* (Wang et al., 2023), *Crassostrea virginica* (Lebordais et al., 2021), and *Monodonta labio* (Li and Han, 2022). Besides, NPs were also found to trigger oxidative stress in *Procambarus clarkia*, *Daphnia pulex* and *Ciona robusta* via transcriptome analysis (Liu et al., 2021a; Capanni et al., 2021; Eliso et al., 2023).

Cephalopoda, as the third-largest and most advanced class of Mollusca, cannot only provide high-quality proteins for humans, but also have scientific research value. Currently, there is no evidence to suggest that NPs have been detected in cephalopods. However, microplastics (MPs, 1 μm–5 mm) have been detected in wild cephalopods *Sepia officinalis* (Oliveira et al., 2020)*, Octopus vulgaris* (Pedà et al., 2022), *Octopus variabilis* (Gong et al., 2021), *Amphioctopus fangsiao* (Yu et al., 2022a), *Dosidicus gigas*, *Abralia veranyi* and *Vampyroteuthis infernalis* (Ferreira et al., 2022). Given that the size of NPs are smaller than MPs, NPs are more easily ingested and difficult to detect. Therefore, NPs may have an impact on cephalopods. However, the toxic molecular mechanisms of oxidative stress caused by NPs to cephalopods are still unexplored.

The golden cuttlefish *Sepia esculenta*, one of the important economic cephalopod species, is distributed mainly in the seas of Russia, China, Singapore, South of Korea, Japan and Philippins (Wang and Zheng, 2017). The *S. esculenta* generally live in the coastal environment that is easily contaminated by plastics (Pedrotti et al., 2016). In this paper, we chose *S. esculenta* larvae to explore the effect of acute NPs exposure on cephalopods. The changes of SOD and MDA enzyme activities revealed that NPs caused oxidative stress to *S. esculenta* larvae. Twenty key genes involved in responding to NPs exposure were obtained by analyzing the transcriptome profiles of *S. esculenta* larvae exposed to NPs for 4 h. The results provide a reference for analyzing organism’s toxic mechanism caused by NPs.

## 2 Materials and methods

### 2.1 *S. esculenta* larvae collection and exposure study

The sexual maturity *S. esculenta* were collected in Qingdao sea area, China, and temporarily raised until laying eggs. The eggs were collected and placed in a breeding pool with flowing seawater and continuously oxygenated during hatching. The eggs hatched after a month and were divided into two groups of 50 individuals in each group. Larvae of control group (C) grew in normal seawater, and exposed group (NPs) larvae grew in seawater with NPs (100 mg/L). Then, the above larvae were collected at 0 h (C_0 h) and 4 h (C_4 h and NPs_4 h) respectively. The collected larvae were stored in liquid nitrogen for future use.

### 2.2 Assay of oxidative stress

The activity of SOD were measured by Total Superoxide Dismutase (T-SOD) assay kit (Hydroxylamine method) purchased from Nanjing Jiancheng Bioengineering Institute. In addition, the MDA levels were detected using Malondialdehyde (MDA) assay kit (TBA method) according to the instructions. Eight replicates were set in each group (C_4h and NPs_4h), and the measured tissue fluid was obtained by grinding 8 randomly selected whole larvae.

The calculation formula for SOD activity is as follows:

$$A=\frac{OD\;value\;\left(contrast\;tube-testing\;tube\right)}{OD\;value\;of\;contrast\;tube}÷50\%\times \frac{V1}{V2}÷C$$

Notes: A, total SOD activity (U/mg prot); V1, total volume of reaction solution (mL); V2, volume of sampling amount (mL); C, protein concentration of testing sample (mg prot/mL).

The calculation formula for MDA level is as follows:

$$L=\frac{OD\;value\;\left(testing\;tube-contrast\;tube\right)}{OD\;value\;\left(standard\;tube-blank\;tube\right)}\times C1÷C2$$

Notes: L, MDA level (nmol/mg prot); C1, concentration of standard substance (10 nmol/mL); C2, protein concentration of testing sample (mg prot/mL).

### 2.3 RNA extraction and sequencing

We used the TRI Reagent method (Rio et al., 2010) with the manufacturer’s protocol to extract total RNA and identified the integrity using Agilent 2100 bioanalyzer (Sodowich et al., 2007). Nine larvae were randomly selected for RNA extraction from groups C_0h, C_4h, and NPs_4h, respectively. Then, the RNA of 9 larvae with equal molar masses in each group was mixed into 3 replicates for subsequent sequencing. Using NEBNext^®^ Ultra™ RNA Library Prep Kit for Illumina^®^ to construct the transcriptome library (Parkhomchuk et al., 2009). Raw reads were sequenced by Illumina NovaSeq 6000 (Illumina, United States), whose SRA accession number were SRR23936172, SRR23936173, SRR23936174, SRR23936175, SRR23936181, SRR23936182, SRR25114243, SRR25114244 and SRR25114245. Removing low quality reads from raw reads to obtain clean reads. The obtained clean reads were mapped to the reference genome (unpublished) using HISAT2.

### 2.4 DEG identification

In this study, the DESeq2 software of R was used as a model to screen differentially expressed genes (DEGs). First, the data were involved in constructing the ddsmodel, after which the dispersion of the samples was estimated using the DESeq function, and finally the differences in gene expression were analyzed. DEGs with *p*-value ≤0.05 to compare groups C_4h and NPs_4h were screened out (Love et al., 2014).

### 2.5 Functional enrichment analyses and network construction

The functional enrichment analysis was performed on DEGs. To ascertain the GO terms and the distribution of DEGs, GO analyses were deployed on the union set distinguished at two distinct time points. Additionally, Gene Set Enrichment Analysis was employed to identify immune-related pathways and genes through the KEGG pathway analysis, thus elucidating the functions of DEGs. Enrichment analyses of GO and KEGG were executed using the DAVID database (https://david.ncifcrf.gov/) 2021 (Jiao et al., 2012). The construction of a protein-protein interaction (PPI) network can offer insights into the correlations amongst oxidative stress pathways, thereby simplifying the identification of pivotal genes. In this study, we leveraged the STRING database (https://cn.string-db.org) to construct a robust PPI network (Szklarczyk et al., 2019).

### 2.6 Quantitative RT-PCR assay

The accuracy of RNA-Seq was verified via qRT-PCR. In this study, 30 hub genes were pinpointed for validation via qRT-PCR. Utilizing Primer Premier 5.0 software, gene primer sequences were formulated based on the spliced transcriptome. The primers related information used in the qRT-PCR is shown in Supplementary Table S1. The gene of *β-actin* is used as housekeeper gene for qRT-PCR due to its evident stability within this experiment. The fluorescence quantification methods implemented were adapted from Liu et al.'s work (Liu et al., 2017).

## 3 Result

### 3.1 Detection of oxidative stress biomarkers

**FIGURE 1:**
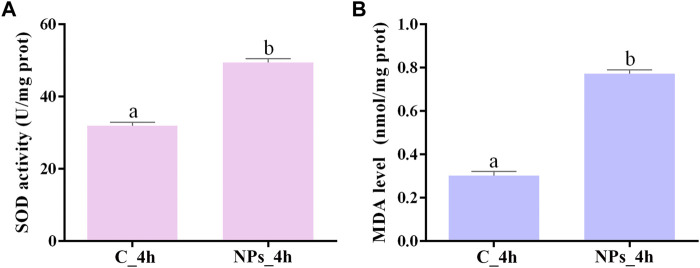
The detection results of SOD activity **(A)** and MDA level **(B)**. The different lowercase letters above the column stand for significant difference between groups (*p* < 0.05).

As shown in Figure 1A, the activity of SOD was higher in the *S. esculenta* larvae exposed to PS-NPs than that in control group (*p*-value = 3.1E-5). The results of RNA-Seq data analysis showed that the transcript levels of SOD were also increased in *S. esculenta* larvae exposed to PS-NPs (Supplementary Table S2). Besides, the level of MDA in *S. esculenta* larvae whole body was increased after PS-NPs exposure (*p*-value = 6.0E-6) (Figure 1B).

### 3.2 Sequencing and analysis of transcriptome

The changes of physiological biomarkers pointed out that the acute exposure of PS-NPs induced oxidative stress in *S. esculenta* larvae. To explore the molecular mechanisms involved, transcriptome sequencing projects were performed. The average of 45,989,845 raw reads per sample were sequenced. Subsequently, after filtering, an average of 44,771,357 clean reads were generated for each sample. The average of Q20 and Q30 were 96.91% and 92.00%, respectively. And the average of GC content in clean reads was 40.24% (Supplementary Table S3). These results suggested a high quality of sequencing.

**FIGURE 2:**
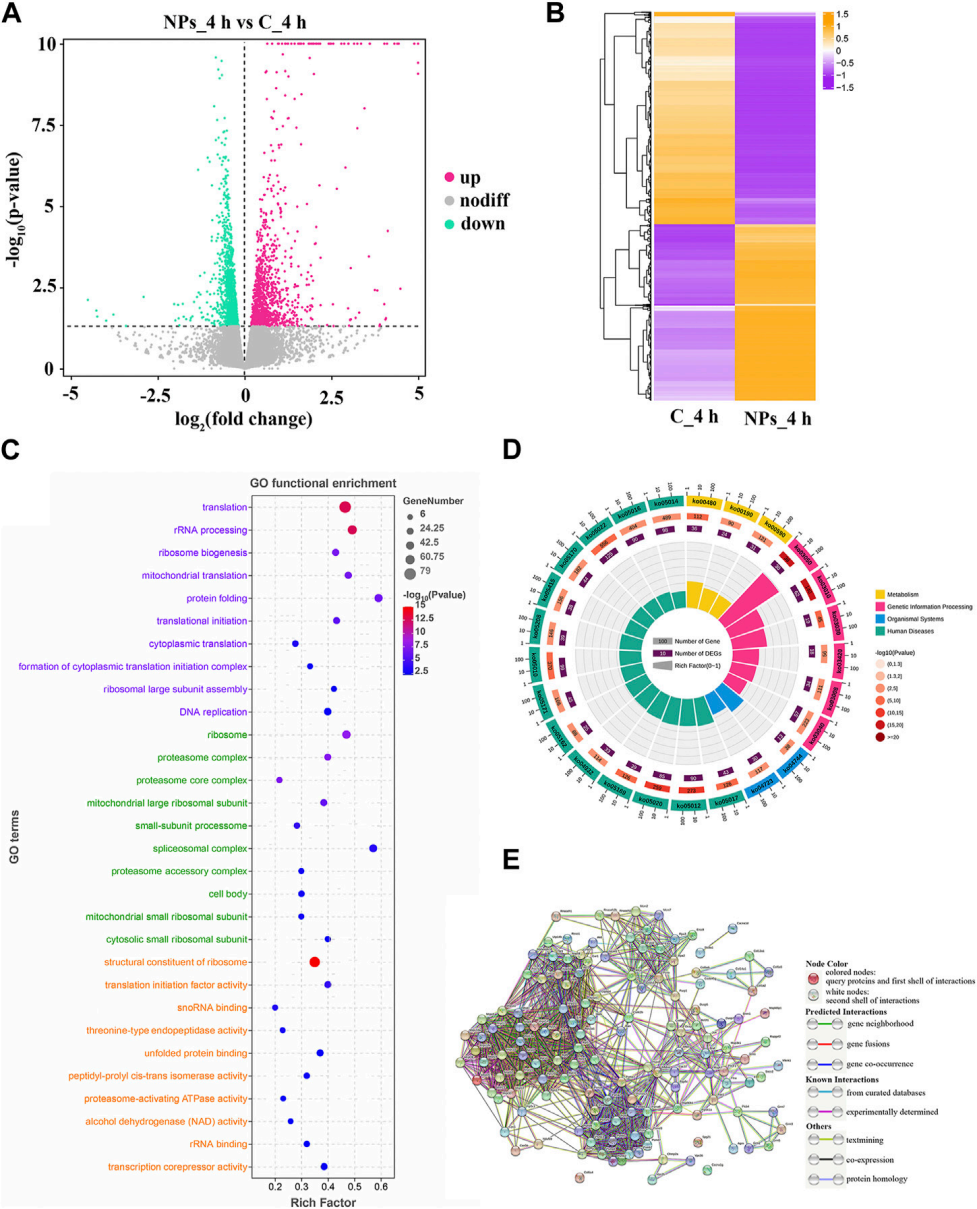
**(A)** Expression of DEGs between C_4h and NPs_4h. Upregulated genes are depicted as rose dots, downregulated genes as medium spring green dots, and non-regulated genes as grey dots. **(B)** Clustering of DEG expression profiles. Each row represents the expression levels of a DEG in each group, while each column represents the overall expression patterns of all DEGs in a group. **(C)** Top 10 significant GO terms. The vertical axis represents GO terms categorized into Biological Process (BP, lavender), Cellular Component (CC, light green), and Molecular Function (MF, orange-yellow). The horizontal axis stands for the rich factor. **(D)** Top 25 level-2 KEGG signaling pathways results. **(E)** PPI network. The circles represent proteins, and the connections between them indicate their interactions, with different connection modes indicating various interaction types.

As the results of differential expression analysis, there were a total of 2570 DEGs (1,166 up- and 1,404 downregulated) at 4 h after PS-NPs exposure (Figure 2A). DEGs expression distribution of all groups was shown in the heatmap, which displayed an obvious difference in the expression pattern of DEGs between the PS-NPs exposure group and non-exposed groups (Figure 2B).

To investigate the function of DEGs, GO and KEGG enrichment analysis were conducted. As shown in Figure 2C, 145 significant GO terms were enriched, and translation, structural constituent of ribosome, and other terms are important for mediating oxidative stress. Based on level-2 KEGG enrichment analysis results, these DEGs were participating in multiple signaling pathways (Figure 2D). The enrichment of 14 KEGG signaling pathways (level-3), such as Ribosome, Proteasome, and MAPK signaling pathway, suggested that PS-NPs exposure have affected multiple biological processes in *S. esculenta* larvae (Table 1).

**TABLE 1:** Significant level-3 KEGG signaling pathways enrichment analysis results.

Pathways	Number of DEGs
Apoptosis	4
Chemical carcinogenesis - reactive oxygen species	6
DNA replication	12
ECM-receptor interaction	4
Endocytosis	5
Growth hormone synthesis, secretion and action	4
MAPK signaling pathway	18
Nucleotide excision repair	9
Phospholipase D signaling pathway	9
PI3K-Akt signaling pathway	5
Proteasome	21
Protein digestion and absorption	6
Ribosome	32
Ribosome biogenesis in eukaryotes	23

Subsequently, 139 DEGs involved in KEGG signaling pathways of Table 1 were used to construct the PPI network to scan hub genes regulating multiple biological processes affected (Figure 2E). The relevant parameters of PPI network are displayed in Supplementary Table S4. Considering the numbers of involved in KEGG signaling pathway and protein interaction, 30 key DEGs were obtained, for instance, RPS5, RPS9 and MRPL4 (Table 2).

**TABLE 2:** Statistics of key genes.

Gene name (abbreviation)	Gene name (official full name)	Number of KEGG signaling pathways	Number of protein–protein interactions
*RPS5*	ribosomal protein S5	1	48
*RPS9*	ribosomal protein S9	1	45
*MRPL4*	mitochondrial ribosomal protein L4	1	44
*MRPS11*	mitochondrial ribosomal protein S11	1	44
*NHP2L1*	small nuclear ribonucleoprotein 13	1	42
*RPL5*	ribosomal protein L5	1	42
*RPL9*	ribosomal protein L9	1	40
*RPS20*	ribosomal protein S20	1	39
*MRPL1*	mitochondrial ribosomal protein L1	1	38
*RPS3A1*	ribosomal protein S3A1	1	37
*MRPL15*	mitochondrial ribosomal protein L15	1	36
*MRPL3*	mitochondrial ribosomal protein L3	1	36
*RPL13A*	ribosomal protein L13A	1	36
*MRPS14*	mitochondrial ribosomal protein S14	1	35
*NFKBIA*	NFKB inhibitor alpha	1	32
*RPL10*	ribosomal protein L10	1	32
*PSMA2*	proteasome 20S subunit alpha 2	1	31
*PSMA3*	proteasome 20S subunit alpha 3	1	31
*MRPS10*	mitochondrial ribosomal protein S10	1	30
*PSMA6*	proteasome 20S subunit alpha 6	1	30
*RPL19*	ribosomal protein L19	1	30
*MRPL12*	mitochondrial ribosomal protein L12	1	29
*GTPBP4*	GTP binding protein 4	1	28
*NMD3*	NMD3 ribosome export adaptor	1	28
*MRPL17*	mitochondrial ribosomal protein L17	1	27
*PSMA8*	proteasome 20S subunit alpha 8	1	27
*MRPL19*	mitochondrial ribosomal protein L19	1	26
*MRPL23*	mitochondrial ribosomal protein L23	1	26
*MRPL32*	mitochondrial ribosomal protein L32	1	26
*PSMA5*	proteasome 20S subunit alpha 5	1	26

### 3.3 Validation of key DEGs

**FIGURE 3:**
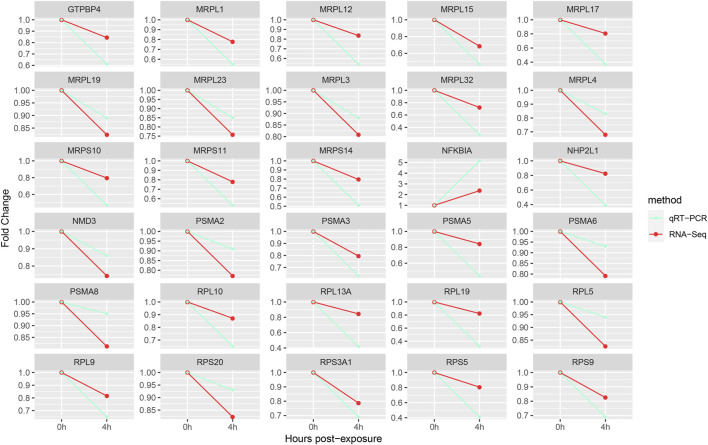
Gene expression verification.

The key DEGs were chosen for quantitative RT-PCR. The results showed the trend of qRT-PCR was consistent with that of RNA-Seq, which suggested that results of transcriptome profile were reliable and accurate (Figure 3).

## 4 Discussion

In recent years, NPs have received increasing attentions as emerging pollutants. Researchers have conducted *in vitro* experiments on various cell types, as well as *in vivo* experiments on model and non-model animals, to study and reveal the toxic effects of NPs (Schröter and Ventura, 2022). However, it is still essential to discuss the toxic effects of NPs in more species. Molluscs, such as bivalves and gastropods, are an important branch in the study of aquatic non-model organisms (Sendra et al., 2021). To enrich the research scope of the effect of NPs on organisms, we selected the *S. esculenta* larvae of Cephalopoda Octopus in Mollusca as the research object. The larvae used in our study have similar size, whose total length is 10.8 ± 0.2 mm and weight is 63.2 ± 8.4 mg. In addition, among various types of waste plastics, PS was chosen because of their higher abundance than others in the coastal sea waters, which also posed a threat to the growth of *S. esculenta* larvae (Pedrotti et al., 2016). Moreover, the smaller the size of the NPs, the easier it is to enter into biological body, even cells. The 50-nm-diameter PS-NPs have been confirmed to cross the intestinal barrier of *Dicentrarchus labrax* after 15 min of exposure (Vagner et al., 2022). Alvarez-Román et al. found that carboxylated PS-NPs (20 nm) could across skin barrier of porcine ear in 2 h (Alvarez-Román et al., 2004). For analyzing the impact of PS-NPs on *S. esculenta* larvae in a short period of time, we selected high concentrations of beads with a diameter of 50 nm. Although the effects of PS-NPs were caused by high concentration, the results can still serve as a reference for further research.

### 4.1 Physiological response

Oxidative stress and even oxidative damage are common in various organisms exposed to NPs, such as mammalian cells, *Danio rerio*, *D. pulex*, and *M. galloprovincialis* (Sarasamma et al., 2020; Liu et al., 2021a; Banerjee and Shelver, 2021; Gonçalves et al., 2022). The occurrence of oxidative stress is triggered by high doses of reactive oxygen species (ROS, like superoxide anion). The removal of ROS in aerobic organisms relies on antioxidant systems. SOD, an important member of the antioxidant enzyme system, has the ability of catalyzing the disproportionation of superoxide anion to hydrogen peroxide, and is used as a biomarker for oxidative stress (Fukai and Ushio-Fukai, 2011). Studies have shown that NPs can enter cells through internalization pathways, causing raise of ROS production and SOD activity, and even directly binding to SOD to alter its activity (Schröter and Ventura, 2022; Wang et al., 2022). In addition, the increase of ROS production could lead to lipid peroxidation. The metabolomics analysis results confirmed that the metabolism of membrane lipids in *Sinonovacula constricta* was affected by acute exposure to PS-NPs (Jiang and Zhang, 2021). MDA, a biomarker of oxidative stress, is an important membrane lipid peroxidation product and can also intensify membrane damage (Sillero-Ríos et al., 2018). After exposure of PS-NPs, SOD activity significantly enhanced, indicating an increase in ROS production. This result is consistent with the improvement of SOD activity in the *Ietalurus punetaus* larvae and in the gills and digestive glands of *Mytilus* spp. caused by acute exposure to NPs (Cole et al., 2020; Jiang et al., 2022). Moreover, the improvement of MDA level suggested the occurrence of membrane lipid oxidation. Research has found that SOD activity and MDA levels also increased in intestine of *A. fangsiao* after exposure to high concentrations of MPs (Zheng et al., 2022). These results indicated that acute exposure to high concentration of PS-NPs can disrupt the redox balance and cause oxidative stress response, which reminded us the hazards of acute exposure to high concentrations of NPs.

### 4.2 mRNA level response

Transcriptome analysis is widely used in the study of molecular level in biology along with the development of sequencing technologies. In recent years, transcriptome analysis has gradually been applied in the toxicology research of NPs on aquatic organisms, such as *B. plicatilis*, *D. pulex*, *Isognomon alatus*, *Mytilus coruscus*, *Cherax quadricarinatus*, *C. robusta*, *D. rerio*, *Oreochromis mossambicus*, *I. punetaus* (Liu et al., 2021a; Pang et al., 2021; Arini et al., 2022; Yu et al., 2022b; Cheng et al., 2022; Jiang et al., 2022; Shin and Jeong, 2022; Eliso et al., 2023; Qi et al., 2023). To research the effect of NPs on the molecular level of *S. esculenta* larvae, we applied RNA-Seq technology in this research.

#### 4.2.1 Translation related signaling pathways and genes

Oxidative stress can influence various biological processes, such as protein synthesis. Proteomic analysis showed that exposure to NPs had an impact on the expression of *D. pulex* proteins (Liu et al., 2021b). The ribosomes, which is composed of ribosomal proteins and ribosomal RNA (rRNA), play a crucial role in cellular protein synthesis. Therefore, the ribosome biogenesis is crucial during the growth and development of organisms. Meanwhile, the ribosome biogenesis, as the most costly cellular process, must be heavily regulated and respond rapidly to stress (such as oxidative stress) or environmental cues (Piazzi et al., 2019). KEGG enrichment analysis of DEGs indicated significant enrichment of Ribosome and Ribosome biogenesis in eukaryotes signaling pathways (Table 1), which is similar to the results of KEGG enrichment analysis in *B. plicatilis* (Shin and Jeong, 2022). This indicates that the ribosome biogenesis of *S. esculenta* larvae did respond to oxidative stress caused by PS-NPs exposure. The eukaryotic ribosome is composed of a large subunit (consisting of 47 Rpls and rRNA of 25S, 5.8S and 5S) and a small subunit (comprising 33 Rpses and 18S rRNA) (Baßler and Hurt, 2019). As shown in Table 2, multiple key genes encoding ribosomal proteins (including *RPL5*, *RPL9*, *RPL10*, *RPL19*, *RPS5*, *RPS9*, *RPS 20* and *RPS3A1*) and mitochondrial ribosomal proteins (containing *MRPL4*, *MRPS11*, *MRPL1*, *MRPL15*, *MRPL3*, *MRPS14*, *MRPS10*, *MRPL12*, *MRPL17*, *MRPL19*, *MRPL23* and *MRPL32*) were screened. In addition, the expression of the above genes was reduced (Figure 2). This suggests that *S. esculenta* larvae respond with oxidative stress induced by PS-NPs exposure by reducing ribosome biogenesis to avoid excessive energy consumption.

#### 4.2.2 Proteasome signaling pathway

Oxidative stress can caused protein oxidation, resulting in protein structure damage. Besides, NPs were reported to interact with proteins to change or even damage protein structures (Hollóczki and Gehrke, 2019; Jiang et al., 2022). To maintain cellular functions, the changed or damaged proteins need to be repaired or removal. The physiological process of protein degradation that consumes vast amounts of energy is mostly regulated by ubiquitin-proteasome system in eukaryotes (Budenholzer et al., 2017). The proteasome is distributed extensively in the cytoplasm and nucleus and is responsible for eliminating damaged proteins (Breusing and Grune, 2008). In *I. punetaus* exposed to PS-NPs, transcriptomic and metabolomics analysis results showed that the response of proteasomes was induced and the contents of energy metabolites were reduced (Jiang et al., 2022). In this study, proteasome signaling pathway was also enriched (Table 1). While the key genes related to proteasome synthesis were downregulated (Figure 3), which indicated that the process of protein degradation was inhibited by reducing the number of proteasome. Additionally, studies have demonstrated that the protein degradation of proteasome mediated decreased at high level of oxidative stress (Breusing and Grune, 2008). These indicate that *S. esculenta* larvae suppress protein degradation process and retain energy by reducing proteasome number to cope with PS-NPs exposure and strong oxidative stress caused.

#### 4.2.3 MAPK signaling pathway

The MAPK signaling pathway plays a critical role in mediating cell functions, for instance, adaptation to various stress. Researches have demonstrated that the MAPK signaling pathway was activated by oxidative stress to trigger inflammation, apoptosis, autophagy, etc (Kim and Choi, 2015). PS-NPs were shown to induce oxidative stress and activate the MAPK signaling pathway in *Monopterus albus* and *Mus musculus* spleen (Tang et al., 2022; Zhu et al., 2023). ROS are considered an important physiological modulator of MAPK signaling pathway. A study found that PS-NPs exposure induced an increase in ROS and activated the expression of genes in the antioxidant system mediated by MAPK-HIF-1/NFκB signaling pathway in *D. pulex* (Liu et al., 2020). In addition, the research showed that NPs exposure caused the activation of components of p38 MAPK signaling pathway in *M. galloprovincialis* hemocytes (Canesi et al., 2016). In *Caenorhabditis elegans*, p38 MAPK signaling pathway was reported to be activated and mediated the protective response to NPs (Qu et al., 2019). And the modulation of genes involved in the MAPK signaling pathway by NPs has been reported in *Paracentrotus lividus* (Della Torre et al., 2014). In the KEGG enrichment analysis results of this study, MAPK signaling pathway was also obtained (Table 1), implying that this signaling pathway played a role in the adaptation to oxidative stress induce by PS-NPs in *S. esculenta* larvae.

## 5 Conclusion

Oxidative stress is a common biological response caused by NPs exposure. In this study, even acute exposure of *S. esculenta* larvae to high concentration PS-NPs could lead to oxidative stress response. In addition, transcriptome analysis showed that translation related ribosome and ribosome biogenesis signaling pathway, protein degradation related proteasome pathway, and adapting to stress related MAPK signaling pathway were obtained. These results provide new references to understand the mechanisms of oxidative stress response induced by NPs.

## Data Availability

The datasets presented in this study can be found in online repositories. The names of the repository/repositories and accession number(s) can be found below: https://www.ncbi.nlm.nih.gov/bioproject/PRJNA947123.
