# Supplementary material for: Transcriptomic analysis of oxidative stress mechanisms induced by acute nanoplastic exposure in Sepia esculenta larvae
Source: Front Physiol. 2023 Aug 8;14:1250513. doi: 10.3389/fphys.2023.1250513 (PMC10442824; doi:10.3389/fphys.2023.1250513)
Supplement: Supplementary file 1 [file Table1.DOCX]

Supplementary Material

Analysis of oxidative stress mechanism induced by acute exposure of nanoplastics based on transcriptome in *Sepia esculenta* larvae

Xiumei Liu^1^, Jianmin Yang^2^, Zan Li^2*^

*** Correspondence:** Zan Li: lizanlxm@163.com

# Supplementary Tables

**Table S1.** List of primers used for quantitative RT-PCR validation.

| Gene name | Forward primer (5’-3’) | TM(°C) | Reverse primer (5’-3’) | TM(°C) | Amplicon length (bp) |
| --- | --- | --- | --- | --- | --- |
| *GTPBP4* | TAAGATCACCCGAGCAGAT | 60 | CAACCTGCCACCGTAAAT | 60 | 101 |
| *MRPL1* | CCTCGCTGTCTTGTGTTATC | 60 | TACATGCTTCGGGCAATG | 60 | 128 |
| *MRPL12* | GAGGAGGACTTGCCTTTATAC | 60 | ATGTGAGGTGCTGCTAATG | 60 | 120 |
| *MRPL15* | GGCTTTGTCAGACACATTAC | 59 | CAAGTAGCCTCGGTTCTTAG | 59 | 115 |
| *MRPL17* | GTTCTGGCACCACGTTATAG | 60 | TGGCCATGGATTTCCTTTC | 60 | 123 |
| *MRPL19* | CGGTTGGATGAGGAATTGT | 60 | GACAGGTACTTTGCTTCCTT | 60 | 108 |
| *MRPL23* | TGTGCCCAAGTACCCTATT | 61 | AGTGGACATGGTTAGAAGGA | 60 | 116 |
| *MRPL3* | AATGTACCTGGCCCAATTC | 60 | GGCAAGTCCTCTGCATAAC | 60 | 113 |
| *MRPL32* | CGTAACTGTTGGAGCTGATG | 61 | CAACTGATCTCCTGGACTTTG | 60 | 107 |
| *MRPL4* | CGACAGACGATCCTGAATAC | 59 | GAGTCCGTAAACAGGCATTA | 60 | 155 |
| *MRPS10* | CGAACCTTTCAGCATCGT | 60 | GTCCCACTTCACTGCATATT | 60 | 144 |
| *MRPS11* | AGCCACAAGGAACAACAC | 60 | GTTCCTTTGCGGGCATTA | 60 | 105 |
| *MRPS14* | GGAGTACGTGGCGTATTAAG | 60 | CAACGAACAGGTTGAGAGAA | 60 | 116 |
| *NFKBIA* | CAGAATGACGAAGGAGATAGC | 60 | CCAAGTGCAAGGGAGTTT | 60 | 151 |
| *NHP2L1* | TTGTTCTGGCTGCTGATG | 60 | CCCAGAGCTTGTTTAGATCG | 60 | 109 |
| *NMD3* | GCAAGTCGCTGAAGTTAGT | 60 | GACATTGGGTCGATCTCTTG | 60 | 130 |
| *PSMA2* | AGGTGTTCGCCCATTTG | 60 | GTAGCCTTCCAAGCAAAGTA | 60 | 114 |
| *PSMA3* | CCGAGAAGAAGCAGCTAAC | 60 | ACAGAGTGTAGGCATGGA | 60 | 101 |
| *PSMA5* | AGGCAGATTGTTCCAAGTAG | 60 | CTCTTCTCTACAGCCAACAC | 60 | 105 |
| *PSMA6* | CACTTGGGTGCTGTATGATT | 60 | CAGCAGCAGTGGCTTTAT | 60 | 108 |
| *PSMA8* | GCAAAGACTGTTCGTGAGTA | 60 | CCAGATTGGACCACTTCTAAC | 60 | 113 |
| *RPL10* | GCCTTCCACTTGCGTATT | 60 | CACCACGCATACCAGTTT | 60 | 103 |
| *RPL13A* | CTCGTGGTCCATTCCATTT | 60 | GTCTACCAAGTGCCTCTTTC | 60 | 105 |
| *RPL19* | CGGTTGGATGAGGAATTGT | 60 | GACAGGTACTTTGCTTCCTT | 60 | 108 |
| *RPL5* | GAATGAAGAGGCATACAGGAG | 60 | GCACGGATTGAGGAATGT | 60 | 105 |
| *RPL9* | ACCCTCTCGCAACTACAT | 60 | GTCACGCCCTTGATCATATT | 60 | 108 |
| *RPS20* | CCATCGCATCCGTATTACTT | 60 | GCACAGGTCCCTTTACTTT | 60 | 113 |
| *RPS3A1* | CGCCCAGGTGAAACTAATC | 60 | GTCAGGAATGAGCTTGTTGA | 60 | 100 |
| *RPS5* | CTCCGGATGATGTTCAAGTC | 60 | CTGAGCCTTACGGAATCTTT | 60 | 125 |
| *RPS9* | TGAGGAACAAGAGGGAAGTA | 60 | GGAGAGCATTACCCTCAAAC | 60 | 123 |

**Table S2.** The FPKM value of oxidative biomarkers.

| Gene Name | C_4h (PFKM) | NPs_4h (FPKM) |
| --- | --- | --- |
| superoxide dismutase (SOD) | 5.449668318 | 6.31515039 |
| catalase (CAT) | 14.61797519 | 15.37753736 |
| glutathione S-transferase (GST) | 11.80780864 | 13.67207731 |
| glutathione peroxidase (GSH-Px) | 475.593297 | 518.6930968 |

**Table S3.** Statistics of sequencing quality.

| Sample | Raw data | Clean data | Q20 (%) | Q30 (%) | GC (%) |
| --- | --- | --- | --- | --- | --- |
| C_0h_1 | 46,113,806 | 44,982,104 | 97.07 | 92.32 | 39.19 |
| C_0h_2 | 50,545,156 | 49,387,700 | 97.44 | 93.02 | 40.39 |
| C_0h_3 | 47,515,546 | 46,359,916 | 97.27 | 92.68 | 39.86 |
| C_4h_1 | 46,179,990 | 45,124,190 | 96.77 | 91.61 | 40.22 |
| C_4h_2 | 46,409,128 | 44,910,160 | 97.27 | 92.62 | 41.01 |
| C_4h_3 | 44,321,476 | 43,052,624 | 96.27 | 90.81 | 40.31 |
| MPs100_4h_1 | 45,433,190 | 44,261,340 | 97.38 | 92.94 | 40.26 |
| MPs100_4h_2 | 42,913,852 | 41,654,616 | 95.51 | 89.48 | 40.58 |
| MPs100_4h_3 | 44,476,460 | 43,209,562 | 97.19 | 92.49 | 40.36 |

**Table S4.** PPI network parameters.

| Network statistics | Number |
| --- | --- |
| Number of nodes | 139 |
| Number of edges | 1,196 |
| Average node degree | 17.2 |
| Clustering coefficient | 0.672 |
| Expected number of edges | 449 |
| PPI enrichment *p*-value | 1.0E-16 |
